# Diagnostic methods to assess inspiratory and expiratory muscle strength*

**DOI:** 10.1590/S1806-37132015000004474

**Published:** 2015

**Authors:** Pedro Caruso, André Luis Pereira de Albuquerque, Pauliane Vieira Santana, Leticia Zumpano Cardenas, Jeferson George Ferreira, Elena Prina, Patrícia Fernandes Trevizan, Mayra Caleffi Pereira, Vinicius Iamonti, Renata Pletsch, Marcelo Ceneviva Macchione, Carlos Roberto Ribeiro Carvalho

**Affiliations:** University of São Paulo, School of Medicine, Hospital das Clínicas, São Paulo, Brazil. Respiratory Muscle Research Group, Pulmonary Division, Instituto do Coração - Incor, Heart Institute - University of São Paulo School of Medicine Hospital das Clínicas, São Paulo, Brazil; University of São Paulo, School of Medicine, Hospital das Clínicas, São Paulo, Brazil. Respiratory Muscle Research Group, Pulmonary Division, Instituto do Coração - Incor, Heart Institute - University of São Paulo School of Medicine Hospital das Clínicas, São Paulo, Brazil; University of São Paulo, School of Medicine, Hospital das Clínicas, São Paulo, Brazil. Respiratory Muscle Research Group, Pulmonary Division, Instituto do Coração - Incor, Heart Institute - University of São Paulo School of Medicine Hospital das Clínicas, São Paulo, Brazil; University of São Paulo, School of Medicine, Hospital das Clínicas, São Paulo, Brazil. Respiratory Muscle Research Group, Pulmonary Division, Instituto do Coração - Incor, Heart Institute - University of São Paulo School of Medicine Hospital das Clínicas, São Paulo, Brazil; University of São Paulo, School of Medicine, Hospital das Clínicas, São Paulo, Brazil. Respiratory Muscle Research Group, Pulmonary Division, Instituto do Coração - Incor, Heart Institute - University of São Paulo School of Medicine Hospital das Clínicas, São Paulo, Brazil; University of São Paulo, School of Medicine, Hospital das Clínicas, São Paulo, Brazil. Respiratory Muscle Research Group, Pulmonary Division, Instituto do Coração - Incor, Heart Institute - University of São Paulo School of Medicine Hospital das Clínicas, São Paulo, Brazil; University of São Paulo, School of Medicine, Hospital das Clínicas, São Paulo, Brazil. Respiratory Muscle Research Group, Pulmonary Division, Instituto do Coração - Incor, Heart Institute - University of São Paulo School of Medicine Hospital das Clínicas, São Paulo, Brazil; University of São Paulo, School of Medicine, Hospital das Clínicas, São Paulo, Brazil. Respiratory Muscle Research Group, Pulmonary Division, Instituto do Coração - Incor, Heart Institute - University of São Paulo School of Medicine Hospital das Clínicas, São Paulo, Brazil; University of São Paulo, School of Medicine, Hospital das Clínicas, São Paulo, Brazil. Respiratory Muscle Research Group, Pulmonary Division, Instituto do Coração - Incor, Heart Institute - University of São Paulo School of Medicine Hospital das Clínicas, São Paulo, Brazil; University of São Paulo, School of Medicine, Hospital das Clínicas, São Paulo, Brazil. Respiratory Muscle Research Group, Pulmonary Division, Instituto do Coração - Incor, Heart Institute - University of São Paulo School of Medicine Hospital das Clínicas, São Paulo, Brazil; University of São Paulo, School of Medicine, Hospital das Clínicas, São Paulo, Brazil. Respiratory Muscle Research Group, Pulmonary Division, Instituto do Coração - Incor, Heart Institute - University of São Paulo School of Medicine Hospital das Clínicas, São Paulo, Brazil; University of São Paulo, School of Medicine, Hospital das Clínicas, São Paulo, Brazil. Pulmonary Division, Instituto do Coração - Incor, Heart Institute - University of São Paulo School of Medicine Hospital das Clínicas, São Paulo, Brazil

**Keywords:** Respiratory muscles, Muscle weakness, Diaphragm, Respiratory function tests, Diagnostic tests, routine

## Abstract

Impairment of (inspiratory and expiratory) respiratory muscles is a common clinical finding, not only in patients with neuromuscular disease but also in patients with primary disease of the lung parenchyma or airways. Although such impairment is common, its recognition is usually delayed because its signs and symptoms are nonspecific and late. This delayed recognition, or even the lack thereof, occurs because the diagnostic tests used in the assessment of respiratory muscle strength are not widely known and available. There are various methods of assessing respiratory muscle strength during the inspiratory and expiratory phases. These methods are divided into two categories: volitional tests (which require patient understanding and cooperation); and non-volitional tests. Volitional tests, such as those that measure maximal inspiratory and expiratory pressures, are the most commonly used because they are readily available. Non-volitional tests depend on magnetic stimulation of the phrenic nerve accompanied by the measurement of inspiratory mouth pressure, inspiratory esophageal pressure, or inspiratory transdiaphragmatic pressure. Another method that has come to be widely used is ultrasound imaging of the diaphragm. We believe that pulmonologists involved in the care of patients with respiratory diseases should be familiar with the tests used in order to assess respiratory muscle function.Therefore, the aim of the present article is to describe the advantages, disadvantages, procedures, and clinical applicability of the main tests used in the assessment of respiratory muscle strength.

## Introduction

Impairment of (inspiratory and expiratory) respiratory muscles is a common clinical finding, not only in patients with neuromuscular disease but also in those with respiratory diseases affecting the lung parenchyma or airways.^(^ 1 ^,^ 2 ^)^

Inspiratory muscle weakness can cause dyspnea^(^ 2 ^)^ and exertion intolerance. However, diagnosis is usually delayed, because most screening protocols for dyspnea do not include assessment of respiratory muscle strength. In addition, when assessment of respiratory muscle strength is performed, it includes tests that yield a high percentage of false negatives, because they depend on patient cooperation (volitional tests). Therefore, for the appropriate investigation and possible confirmation of respiratory muscle weakness as a cause of respiratory failure, it is of paramount importance to be familiar with non-volitional measures and even with techniques that are more invasive, such as the measurement of transdiaphragmatic pressure.

The aim of the present article is to describe the advantages, disadvantages, procedures, and clinical applicability of the main diagnostic methods to assess respiratory muscle strength. Although we discuss the main measures of respiratory muscle strength, we do not specifically address the diagnosis of muscle fatigue. We also do not describe other related tests that are not specific or sensitive for confirming the diagnosis of respiratory muscle weakness, such as spirometry and arterial blood gas analysis.

## Volitional tests for measuring inspiratory muscle strength

### Maximal inspiratory pressure

Maximal inspiratory pressure (MIP) is the most widely used measure of respiratory muscle strength in patients with suspected respiratory muscle weakness.^(^ 3 ^)^ It is determined by measuring upper airway pressure (mouth for outpatients and trachea for intubated or tracheostomized patients) during a maximal voluntary inspiratory effort. The measured pressure is a composite of the pressure generated by the inspiratory muscles and the elastic recoil pressure of the lungs and chest wall.

Advantages

It uses low-cost, portable equipment; it is easy and rapid to perform; it is noninvasive; and it has well-established reference values, in different populations (lower limit of normal of 60 cmH_2_O for females and 80 cmH_2_O for males).^(^ 4 ^-^ 6 ^)^ In addition, since the relationship between lung volumes and inspiratory muscle strength is not linear,^(^ 7 ^)^ the measurement of MIP can diagnose inspiratory muscle weakness earlier than would be possible based on changes in lung volumes.

Disadvantages

The maneuver is not intuitive and depends on patient cooperation. Therefore, a low value might not mean weakness, but rather a lack of cooperation. MIP has high coefficients of intraindividual and interindividual variation (10 to 13%)^(^ 8 ^)^ and low accuracy for predicting successful extubation in mechanically ventilated patients.^(^ 9 ^)^

How to measure

MIP is measured from RV or from functional residual capacity (FRC). Since there is an inverse relationship between lung volume and inspiratory muscle strength,^(^ 6 ^)^ measurements from RV yield module values that are 30% higher than those obtained from measurements from FRC. Although measurements from RV yield higher values, some physicians and researchers use measurements from FRC because they more reproducible and more easily performed by patients. However, when measurements from FRC are made, it is necessary that FRC volume be known, because this volume will affect the pressure generated.

**Figure 1 -:**
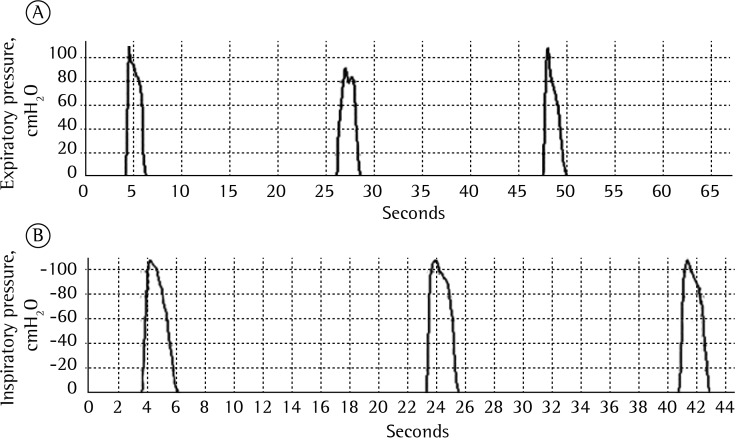
Measurement of MEP and MIP with a digital pressure manometer (model MVD 300; Globalmed, Porto Alegre, Brazil). In A, positive MEP values. In B, negative MIP values.

The measurement of MIP can be made with an analog or digital pressure manometer.^(^ 10 ^)^ Digital devices are preferred over analog devices, given that the highest MIP value occurs briefly and may go unnoticed on an analog display (Figure 1). Measurements are usually made with patients in a sitting position, with or without nose clips. Patients are asked to exhale to RV and then perform a maximal inspiratory effort, sustaining it for 1 to 2 seconds. To prevent overestimation of values because of glottal closure and pressure by the mouth muscles, there should be a 2-mm-wide opening in the mouthpiece, which can be a rigid tubular mouthpiece or a rubber mouthpiece. The latter gives slightly higher values.^(^ 11 ^)^

**Figure 2 -:**
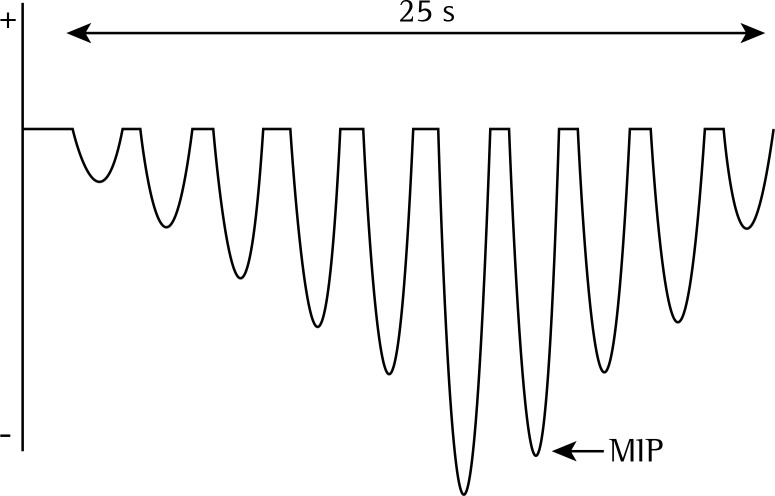
Variation in inspiratory pressure during measurement of MIP with a one-way valve. The highest value usually occurs within 15 to 20 seconds.

In critically ill intubated patients who are uncooperative, the optimal measurement of MIP is made with a one-way valve (it permits exhalation, but occludes during inspiration) attached to the tube and takes 25 seconds (Figure 2).^(^ 12 ^)^

In any method, the maneuver should be repeated 3 to 8 times, and the highest value recorded will be used for analysis. The reproducibility of the MIP measurement, with or without a one-way valve, is 10%. 

Clinical applicability

Determining MIP is important in the diagnosis of inspiratory muscle weakness, which can occur in pulmonary, cardiac, and neuromuscular diseases. In addition, the measurement of MIP can aid in the differential diagnosis of dyspnea^(^ 2 ^)^; in the differential diagnosis of obstructive lung disease of unknown origin; in assessing response to cardiopulmonary physiotherapy and rehabilitation; in prescribing and monitoring respiratory muscle training^(^ 13 ^,^ 14 ^)^; and, in critically ill patients, in assessing the possibility and success of weaning from mechanical ventilation.^(^ 10 ^)^

### Sniff nasal inspiratory pressure

The search for a method for the measurement of inspiratory muscle strength that would overcome the limitations of MIP, as well as being noninvasive (avoiding the need for an esophageal balloon), resulted in the proposal of measuring nasal inspiratory pressure during a sniff.^(^ 15 ^)^ SNIP is an acronym for sniff nasal inspiratory pressure. SNIP measures the joint activity of the diaphragm and other inspiratory muscles and accurately reflects esophageal pressure (Pes), having the advantage of being noninvasive.^(^ 15 ^,^ 16 ^)^ However, the correlation between Pes and SNIP is reduced when there is significant airway obstruction, which occurs in asthma and COPD. Electromyographic studies have shown that, during SNIP, there is selective contraction of the muscles involved in breathing, especially the inspiratory accessory muscles, which demonstrates the specificity of the test.^(^ 17 ^)^

Although SNIP has a reasonable correlation with MIP,^(^ 18 ^)^ the former does not replace the latter and should be used as an additional measure in the assessment of inspiratory muscle strength, because the use of only one test can overestimate muscle weakness, whereas the use of both tests reduces the rate of false-positive results for respiratory muscle weakness by nearly 20%.^(^ 18 ^,^ 19 ^)^

Advantages

It uses pressure manometers, which are simple and inexpensive equipment that also measures MIP; it is easy to perform, because it is based on an intuitive maneuver, which makes the measurement more reproducible; and it has well-established normal values, in different populations (lower limit of normal of 60 cmH_2_O for females and 70 cmH_2_O for males; Table 1).^(^ 20 ^-^ 24 ^)^

**Table 1 -:** Lower limits of normal for respiratory muscle strength tests.a

Method	Lower limit of normal
MIP (cmH_2_O)	60 (F) / 80 (M)
MEP (cmH_2_O)	120 (F) / 150 (M)
SNIP (cmH_2_O)	60 (F) / 70 (M)
Sniff Pes**(cmH_2_O)	60 (F) / 70 (M)
Sniff Pdi**(cmH_2_O)	70 (F) / 80 (M)
Twitch Pes (cmH_2_O)	12 (F and M)
Twitch Pdi (cmH_2_O)	20 (F and M)
Twitch Pga (cmH_2_O)	16 (F and M)
Cough Pga (cmH_2_O)	95 (F) / 130 (M)
Twitch Pga at T10 (cmH_2_O)	16 (F and M)
Diaphragm motion on US – breathing at rest (mm)	11
Diaphragm motion on US – deep breathing (mm)	47
Diaphragm thickening on US – breathing at rest (mm)	1.5
Rate of thickening during inspiration to TLC on US	20%

F: female; M: male; SNIP: sniff nasal inspiratory pressure; Pes: esophageal pressure; Pdi: transdiaphragmatic pressure; Pga; gastric pressure; and US: ultrasound.

a Modified from Polkey & Moxham.(21).

Disadvantages

It depends on patient cooperation, it cannot be used in mechanically ventilated patients, it can underestimate values in patients with marked airway obstruction, and it should be used with caution in those with nasal obstruction.

How to measure

The maneuver can be performed in any body position (the most common is sitting), because, despite minor variations, changes in body position do not result in significant changes in SNIP. ^(^ 20 ^)^ One nostril should be completely closed by a nose plug to prevent pressure from leaking, whereas the other nostril should be absolutely patent. After a period of quiet breathing, the maneuver begins with a fast deep inspiration from FRC and the mouth closed. A firm verbal command is needed, given that the maneuver should be short (≤ 500 ms) and explosive so that it causes the collapse of the unplugged nostril. Ten maneuvers should be performed. However, if there is a considerable increase in the values obtained in the last maneuvers, up to ten more maneuvers can be performed. Twenty maneuvers are also necessary when the values of the first ten maneuvers are below predicted values and when inspiratory muscle weakness due to exertion is suspected, such as in neuromuscular diseases. ^(^ 26 ^)^ The highest value recorded in this series of maneuvers is used for analysis.

Clinical applicability

SNIP is very useful in assessing inspiratory muscle strength and has high specificity^(^ 17 ^)^ compared with MIP. In recent years, SNIP has been used for diagnosis and monitoring of muscle weakness in various pathologies in which a deficit in inspiratory muscle strength is part of the natural history of the disease, such as in neuromuscular^(^ 23 ^)^ and pulmonary^(^ 25 ^)^ diseases.

### Inspiratory mouth pressure

Inspiratory mouth pressure (Pm) is measured with a pressure sensor attached to a mouthpiece (as in the measurement of MIP) or to a tracheal tube.^(^ 27 ^)^ It is usually used in three clinical situations. First, it is used as an indirect measure of Pes during a sniff, when esophageal catheters are not available or when esophageal catheter placement is not possible. In this situation, a limitation of Pm is that, for patients, its measurement is more difficult than that of SNIP.^(^ 3 ^)^ Its second use is in ascertaining the correct placement of the esophageal catheter, which is discussed later. Finally, Pm is also used in the measurement of P_0.1_, which is the pressure generated in the first 100 ms of an inspiratory effort against a closed airway, and it correlates better with the measurement of respiratory drive than with the measurement of MIP.

Advantages

It is a simple, noninvasive method, it can use the same instruments used in the measurement of MIP and SNIP, and it is an alternative method in patients with contraindication to esophageal catheters (esophageal varices or severe hypoxemia) or in patients in whom an esophageal catheter cannot be placed (intolerance to passage of catheters or airway anatomical changes).

Disadvantages

For patients, measurements made through the mouth are more difficult than nasal measurements, and the former provide no additional advantages over the latter. As occurs with MIP, Pm does not differentiate between which respiratory muscle is affected. In patients with severe expiratory flow limitation and parenchymal disease, the transmission of pressure along the airways may be affected, and, in such cases, Pm may not be an accurate measure of alveolar pressure.^(^ 3 ^)^ As occurs with MIP, values can be affected by the type of mouthpiece used.^(^ 11 ^)^

How to measure

In the measurement of Pm, the cross-sectional area of the mouthpiece should be wide enough to prevent errors arising from the Bernoulli effect (a reduction of the cross-sectional area of a tube leads to an increase in the speed of the gas flow and a decrease in pressure). In addition, the compliance of the cheeks can distort the measurement, and, to work around this limitation, the cheeks need to be supported by both hands during the measurement. In the measurement of P_0.1_, the distal end of the mouthpiece should be closed for verification of the correct positioning of the esophageal balloon.^(^ 27 ^)^ Pm can be measured nonvolitionally as well, by means of phrenic nerve stimulation,^(^ 28 ^-^ 30 ^)^ a topic that is discussed further below.

Clinical applicability

**Figure 3 -:**
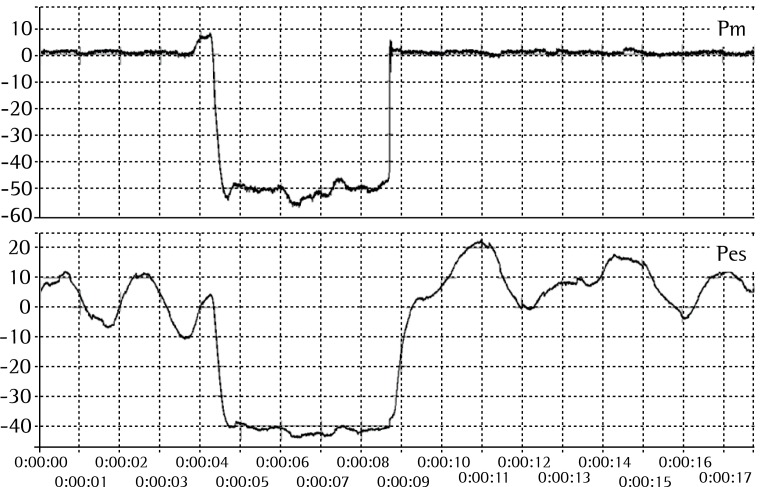
Comparison between inspiratory mouth pressure (Pm) and inspiratory esophageal pressure (Pes) during mouth occlusion (Baydur maneuver), for ascertaining the correct location of the esophageal catheter. Note the good correlation between the two measurements.

It is mainly used as an indirect measure of Pes during a sniff,^(^ 1 ^)^ in order to confirm inspiratory muscle weakness^(^ 2 ^)^; and for verifying the correct positioning of the esophageal balloon (Figure 3) by using the Baydur test^(^ 27 ^,^ 31 ^)^-see *How to measure* in the next item.

### Transdiaphragmatic pressure

**Figure 4 -:**
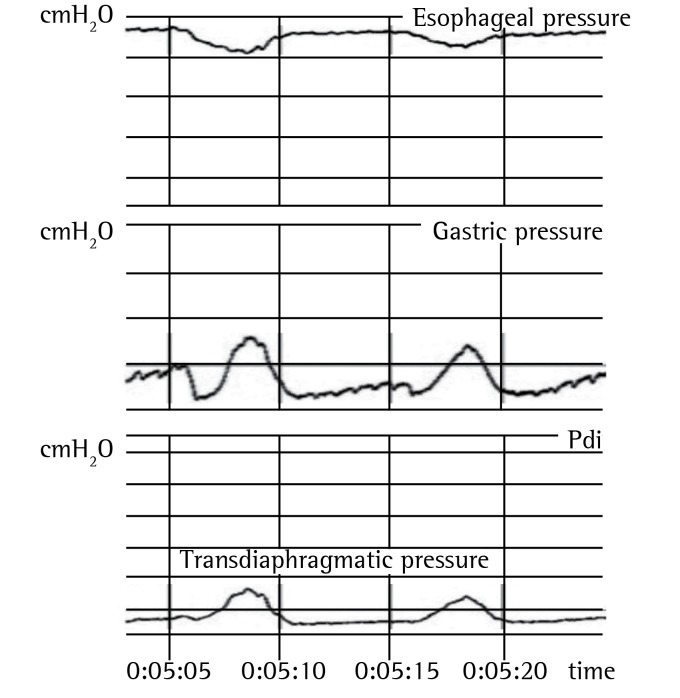
Transdiaphragmatic pressure. The top curve represents esophageal pressure, the middle curve represents gastric pressure, and the bottom curve represents transdiaphragmatic pressure (Pdi). In this example, there are differences in the esophageal and gastric pressure measurement range.

Transdiaphragmatic pressure (Pdi) is the difference between gastric pressure (Pga) and Pes (Pdi = Pga − Pes; Figure 4) and translates the force generated by the diaphragm rather than by the other respiratory muscles. 

Advantages

The strength of the diaphragm, which is the main inspiratory muscle, being responsible for 60 to 70% of the tidal volume in normal breathing, has well-established reference values in volunteers of different groups (lower limit of normal of 70 cmH_2_O for females and 80 cmH_2_O for males during a sniff),^(^ 32 ^)^ as well as in patients with different respiratory diseases (Table 1).^(^ 33 ^,^ 34 ^)^

Disadvantages

It is an invasive method, which depends on passing catheters through the nose into the distal esophagus and stomach and which uses materials that are not readily available in most public hospitals. It depends on an experienced examiner for correct placement of the catheters.

How to measure

Pdi can be measured with air-filled latex balloon catheters, fluid-filled catheters, or microtransducer catheters.^(^ 35 ^,^ 36 ^)^ The use of balloon catheters requires passing a catheter into the esophagus and another one into the stomach, although a catheter with two balloons, which prevents the need for a second catheter, has recently been placed on the market.^(^ 37 ^)^ The microtransducer catheter makes Pes and Pga measurements with only one catheter, as well as having the advantage of being better tolerated by patients and having a fast response time, which ensures more accurate measurements in fast maneuvers,^(^ 38 ^)^ such as measurements using magnetic stimulation of the phrenic nerve. 

**Figure 5 -:**
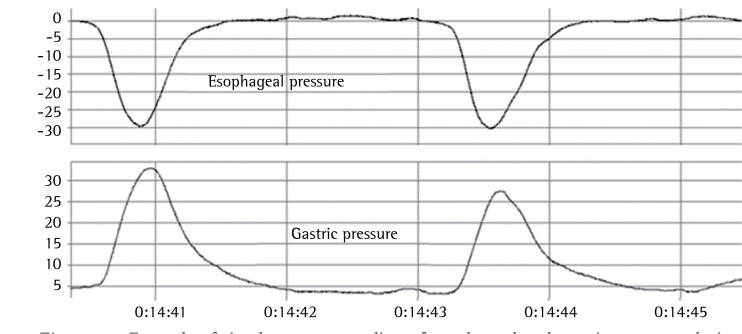
Example of simultaneous recording of esophageal and gastric pressures during forced inspiration. Note that as esophageal pressure becomes more negative, gastric pressure becomes positive, creating a mirror image of the two curves.

When using latex balloon catheters, which are the most common ones, one catheter is placed into the distal esophagus and one is placed into the stomach. To ensure correct positioning, it is necessary to observe the Pes and Pga curves. This is easy because, during inspiration, Pes becomes negative and Pga becomes positive, in a mirror image (Figure 5). The final step to ensure that the Pes detected by the balloon catheter is correct is to compare it with the Pm measured by using the closed mouthpiece. If the esophageal positioning is correct, that is, if it reflects pleural pressure well, the variation in Pes will be at least 80% of the variation in Pm. This confirmatory test is known as the Baydur test^(^ 27 ^)^ and has been validated for different lung volumes and postural positions.^(^ 31 ^)^

Pdi can be measured during normal breathing or during maximal inspiratory maneuvers, usually during a sniff. In addition, Pdi can be measured during magnetic stimulation of the phrenic nerve, which is discussed in Non-volitional tests for measuring inspiratory muscle strength (see Electrical and magnetic phrenic nerve stimulation).

Clinical applicability

Because it is an invasive method that requires complex equipment and is complex to perform, it is used almost exclusively to determine respiratory muscle strength.^(^ 35 ^)^ Its main use is to enable a more representative measurement of diaphragm strength, especially in patients with airway obstruction, in whom Pes would not be accurately reflected by Pm or by SNIP.^(^ 3 ^,^ 18 ^)^

## Non-volitional tests for measuring inspiratory muscle strength

### Electrical and magnetic phrenic nerve stimulation

**Figure 6 -:**
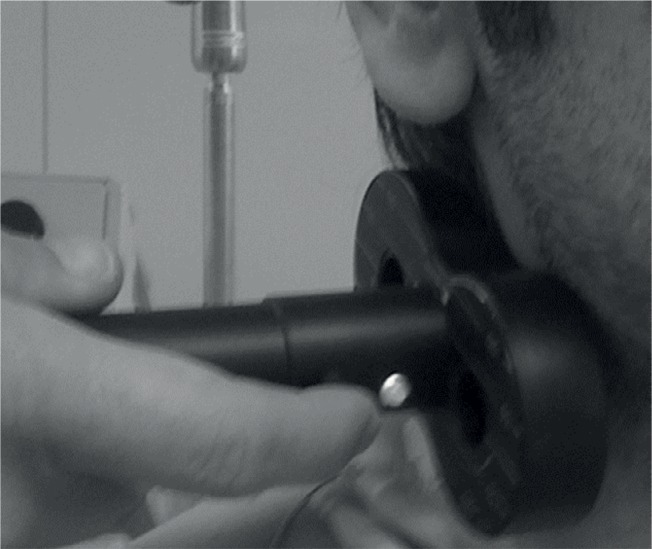
Phrenic nerve magnetic stimulation coil placed on the anterior cervical area of a volunteer.

The use of non-volitional tests for measuring inspiratory muscle strength is recommended when patients have difficulty understanding or performing the maneuvers, generating low values during the volitional maneuvers (MIP, SNIP, or Pm); or when there is considerable variation in the measurements, which is probably secondary to different levels of effort. To obtain maximal involuntary inspiratory contraction, there are two possible methods that yield similar results: electrical stimulation or magnetic (twitch) stimulation of the phrenic nerve. Both are based on stimulating the cervical phrenic nerve, which is superficial (Figure 6). Electrical stimulation is painful, and there are reports of it inducing convulsion; however, it is more specific for diaphragm stimulation than is magnetic stimulation.^(^ 39 ^)^ Magnetic phrenic nerve stimulation causes minimal discomfort, which is well tolerated by most patients.^(^ 40 ^)^ Its principle is to create a magnetic field in the cervical region by placing small coils over this region. Pdi values are similar with magnetic and electrical stimulation.^(^ 40 ^)^ Since magnetic phrenic nerve stimulation provides greater safety and comfort, its use has surpassed that of electrical stimulation.

Advantages

It allows the measurement of inspiratory muscle strength, irrespective of patient cooperation or understanding. The reason for it is that the diaphragm is innervated exclusively by the phrenic nerve, and this enables overall muscle stimulation. Magnetic stimulation easily penetrates tissues and bones, preferentially activating larger neural fibers rather than smaller fibers, which are responsible for mediating pain.^(^ 41 ^)^ There are well defined Pdi values after bilateral cervical magnetic stimulation (lower limit of normal of 20 cmH_2_O for females and males).^(^ 40 ^)^

Disadvantages

Since the magnetic field can stimulate other cervical nerves and muscles, its use is usually less specific for the measurement of diaphragm strength than is that of electrical stimulation, although this difference does not appear to be clinically relevant.^(^ 39 ^,^ 40 ^)^ Another disadvantage is that magnetic stimulation equipment is not readily available and is costly.

How to measure

The device consists of a base with a capacitor connected to a coil that is placed over the site to be stimulated. The type of coil has considerable influence on the intensity and form of the magnetic field generated. Initially, the most widely used coil was the 90-mm circular coil placed on the posterior cervical area at the level of the seventh cervical vertebra. This coil, however, created a larger magnetic field and ended up stimulating other neural fibers in the neck and upper intercostal muscles. The coil currently in use is the 45-mm figure-of-eight coil, which creates a field more focused on the phrenic nerve when it is placed over the posterior edge of the sternocleidomastoid muscle at the level of the cricoid cartilage. With one figure-of-eight coil, it is possible to measure the force generated by one hemidiaphragm alone, and with two coils activated simultaneously, it is possible to measure the force generated by both hemidiaphragms together.^(^ 3 ^,^ 39 ^,^ 40 ^)^ The measurements most commonly made using magnetic phrenic nerve stimulation are those of Pdi and Pm. Although the measurement of Pm is noninvasive, it depends on the glottis remaining open after magnetic stimulation, and this may result in considerably underestimated values in patients with increased airway resistance and severe parenchymal disease. A disadvantage of measuring Pdi is the need for insertion of esophageal and gastric catheters.

Clinical applicability

It is used mainly in research and clinical settings when one wants to avoid variability related to patient cooperation or when patients cannot cooperate properly, such as mechanically ventilated patients or those who cannot understand or perform the requested maneuvers.

### Diaphragm ultrasound

In recent years, there has been a great increase in interest in the use of ultrasound to assess the diaphragm.^(^ 42 ^)^ The literature has demonstrated that diaphragm ultrasound is a useful tool for bedside assessment, because it is noninvasive and radiation-free, it is readily available in hospitals, and it allows repeated assessments. Diaphragm ultrasound has accuracy similar to that of fluoroscopy for assessment of diaphragm motion.^(^ 43 ^)^ There have been some recent articles in different publications discussing the use of ultrasound for assessment of diaphragm function in mechanically ventilated patients, especially for predicting extubation failure,^(^ 44 ^)^ and for diagnosis and monitoring of inadvertent injury occurring during surgery.^(^ 45 ^)^ There have also been articles discussing the use of ultrasound for diagnosis and monitoring of diaphragm paralysis in outpatients. ^(^ 46 ^,^ 47 ^)^

Advantages

It is a noninvasive, radiation-free method; it can be repeated several times over a short period of time; it uses a basically configured ultrasound system, which is a piece of equipment that has become common in hospitals and clinics; and the learning time is not long. Finally, normal values for diaphragm thickening and motion are well established (Table 1). For males and females, diaphragm motion during quiet breathing should be at least 11 mm, and during deep breathing, it should be at least 47 mm. For males and females, diaphragm thickening after inspiration to TLC should be at least 1.5% or 20%.^(^ 48 ^-^ 50 ^)^

Disadvantages

It is an operator-dependent method, and, in obese patients with abdominal distention or extensive dressings, it can be difficult to obtain good quality images. Diaphragm motion is affected by abdominal pressure and contents, and this decreases the relationship between diaphragm motion and variation in lung volume during the maneuvers, thus requiring the concomitant use of a pneumotachograph.

How to measure

**Figure 7 -:**
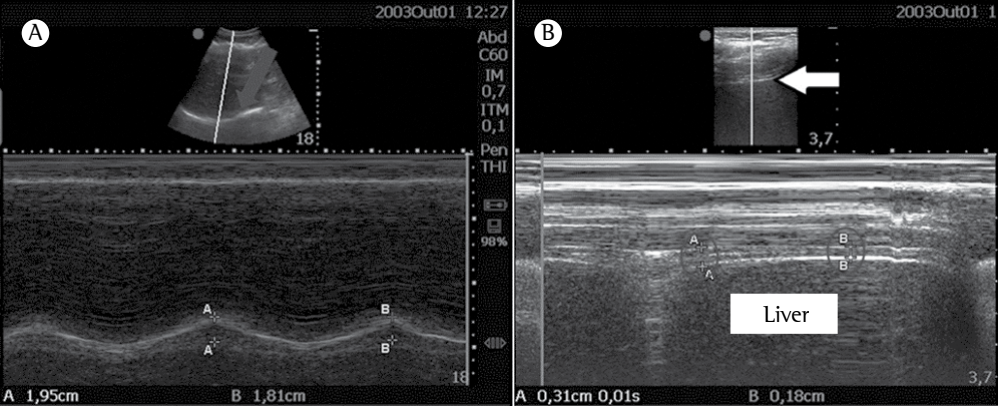
Ultrasound imaging of the diaphragm. In A, an ultrasound scan for assessment of diaphragm motion. The top image is a B-mode image, and the gray arrow indicates the diaphragm, which is seen as a more echogenic line. The bottom image is the top image in M-mode and serves to measure diaphragm excursion (distances between A-A and B-B points) during breathing at rest. In A, diaphragm motion was 19.5 and 18.1 mm and was, therefore, normal. In B, an ultrasound scan for assessment of diaphragm thickening. The top image is a B-mode image, and the white arrow indicated the diaphragm, which is seen as a more echogenic line. The bottom image is the top image in M-mode and serves to measure diaphragm thickening during inspiration (A-A points) and the next expiration (B-B points). In B, diaphragm thickening was 1.3 mm and was, therefore,

The use of ultrasound allows us to measure diaphragm dome motion and diaphragm thickness in the zone of apposition to the rib cage (Figure 7A and B). Diaphragm dome motion is measured with a (cardiac or convex) low-frequency (3-5 MHz) transducer, which is held against the highest point of the diaphragm (the diaphragm dome). Depending on the method used, the transducer can be placed in the transverse^(^ 50 ^)^ or longitudinal^(^ 49 ^)^ direction in the subcostal region, the reference being the point between the midclavicular and anterior axillary lines. The diaphragm is visualized in B-mode, and diaphragm excursion is measured in M-mode, which reduces interobserver variability. ^(^ 51 ^)^ It is important to assess motion not only during normal breathing but also during fast and slow deep inspiration. Assessing motion during a sniff is useful because it enhances the detection of paradoxical motion of the diaphragm, which may not occur during normal breathing. Motion measurement of the right and left hemidiaphragms yields equal values, but measurement on the right is easier because of the presence of the liver, which creates an acoustic window (Figure 7A).

Diaphragm thickening is measured with a high-frequency (7-10 MHz) transducer placed in the zone of apposition of the diaphragm to the midaxillary line. Diaphragm thickness is the distance between the two hyperechogenic lines representing its borders (Figure 7B). This thickness is usually measured at FRC and also at TLC after a maximal inspiration.^(^ 48 ^)^ Diaphragm thickening should increase by at least 20% at TLC when compared with the value obtained at FRC.

Clinical applicability

Diaphragm ultrasound can be used at the bedside or in an outpatient setting. It allows the assessment of two useful fundamental parameters: diaphragm motion and diaphragm thickness. In addition, it can be performed in different body positions. The respiratory diseases about which there exist the largest number of studies with the use of diaphragm ultrasound are COPD and diaphragm paralysis, and there are studies with the use of diaphragm ultrasound in weaning from mechanical ventilation.^(^ 47 ^,^ 48 ^)^

## Volitional tests for measuring expiratory muscle strength

### Maximal expiratory pressure

Maximal expiratory pressure (MEP) is the most widely used measure of expiratory muscle strength in critically ill patients and in outpatients.^(^ 3 ^)^

Advantages

It is simple and rapid to perform; it uses low-cost, low-complexity equipment (the same used for measuring MIP); and it has well-established reference values (lower limit of normal of 120 cmH_2_O for females and 150 cmH_2_O for males; Table 1).^(^ 52 ^)^

Disadvantages

It depends on patient cooperation and on the coordination between the patient and the examiner, as well as having low accuracy for predicting cough capacity. It has a high rate of false-positive results for expiratory muscle weakness because it can overestimate the number of patients with expiratory muscle weakness, given that low values are caused by submaximal efforts or air leaks around the mouthpiece, which is common in patients with facial muscle weakness.

How to measure

MEP is measured with a pressure manometer. Measurements are usually made with patients in a sitting position and with a nose clip, although the use of a nose clip is not necessary. MEP can be measured from TLC or from FRC. Patients perform a maximal expiratory effort and sustain it for 1 to 2 seconds. The maneuver should be repeated 3 to 8 times, and the highest value recorded is used for analysis. Since there is a direct relationship between lung volume and expiratory muscle strength,^(^ 51 ^)^ measurements from TLC yield higher values than those obtained from measurements from FRC.

Clinical applicability

Its major use is in assessing cough strength, given that one of the phases of cough is explosive expiration and expiratory muscle weakness correlates with respiratory infections^(^ 53 ^)^ and extubation failure.^(^ 54 ^)^

### Cough gastric pressure

Measuring Pga during a cough is a useful additional test in the assessment of expiratory muscle weakness, because abdominal muscles are the primary muscles responsible for expiratory flow.

Advantages

It has well-established reference values (lower limit of normal of 95 cmH_2_O for females and 130 cmH_2_O for males; Table 1),^(^ 55 ^)^ and its specificity is greater than that of MEP. Therefore, the negative predictive value of cough Pga is higher than that of MEP alone. A previous study showed that 42% of patients with reduced MEP actually had normal cough Pga values.^(^ 55 ^)^

Disadvantages

Its main disadvantage is that it is an invasive method that requires the insertion of a catheter with a pressure sensor into the stomach.

*How to measure* Pga is typically measured with a catheter, following the same recommendations as those already described for the measurement of Pdi (*see Transdiaphragmatic pressure*). Patients in a sitting position are instructed to inhale to TLC and then cough with maximum force, repeating the maneuver at 30-second intervals until the values stop increasing. Typically, up to six maneuvers are necessary.^(^ 55 ^)^

Clinical applicability

It is used to rule out expiratory muscle weakness, especially in patients suspected of having reduced cough strength due to expiratory pump impairment, such as patients with neuromuscular disease and critically ill patients immediately before or after extubation.

## Non-volitional tests for measuring expiratory muscle strength

### Gastric pressure after magnetic stimulation of the anterior abdominal wall muscles

In uncooperative patients, expiratory muscle strength can be assessed by measuring Pga after neural magnetic stimulation of the abdominal wall muscles.^(^ 3 ^,^ 56 ^,^ 57 ^)^ Magnetic stimulation is produced by placing a circular coil over the dorsal spine, at the level of the eighth to tenth thoracic vertebra (T8 to T10).^(^ 56 ^,^ 58 ^)^

Advantages

Values are independent of patient cooperation.

Disadvantages

This measurement is invasive because it requires passage of a catheter into the stomach, and there exists only one study that reported reference values, in a small sample of individuals (lower limit of normal of 10 cmH_2_O for females and males; Table 1).^(^ 18 ^)^

How to measure

This measurement can be made with a balloon catheter or a microtransducer catheter placed into the stomach. To ensure the correct positioning of the catheter, it is necessary to observe the pressure curve, which, during inspiration, should have positive values (Figure 4). Another means to ensure correct positioning in the stomach is to employ manual compression of the epigastrium and observe an increase in Pga. When the catheter is correctly positioned in the stomach, magnetic stimulation is performed over the dorsal spine, between T8 and T10, and the pressure variation is recorded. Approximately 5 measurements are performed at intervals of at least 30 seconds to avoid muscle potentiation.

Clinical applicability 

This measurement is used to confirm possible expiratory muscle weakness,^(^ 43 ^,^ 59 ^)^ especially in individuals with difficulty performing volitional tests intended to assess cough strength, such as patients with neuromuscular disease and critically ill patients.

### Electromyography

Electromyography is the study of muscle activity based on analysis of electromyographic signals, which are electrical manifestations generated during voluntary or stimulated contractions. It can performed with electrodes attached to the skin (surface electromyography)^(^ 59 ^)^ or with fine needles inserted into the surface of the muscle that is assessed (needle electromyography).^(^ 60 ^,^ 61 ^)^ In the case of respiratory muscles, there is a third option, which is the use of esophageal electrode catheters to perform crural diaphragm electromyography.^(^ 62 ^,^ 63 ^)^

Advantages 

Surface electromyography is a noninvasive, easy-to-use method, being quite useful for continuous monitoring. It is extremely sensitive for detecting muscle contractions.^(^ 64 ^)^ Needle electromyography is minimally invasive and mildly painful.

Disadvantages

The major problem in performing surface electromyography is interference from the activity of other muscle groups (cross-talk). Because electromyography is highly sensitive, it is often difficult to isolate the activity of only one muscle group. Another disadvantage is the limited standardization for analysis of the signal, which can be interpreted visually through its amplitude and duration components or through a numerical value obtained by squaring the signal amplitude and subsequently extracting the root square of the result (root mean square value). Diaphragm electromyography with esophageal catheters is invasive and depends on materials and skills that are highly specific and are still not readily available, although there is a mechanical ventilation apparatus available on the market that has a ventilation mode based on the acquisition of esophageal electromyography signals (the Neurally Adjusted Ventilatory Assist [NAVA] mode of ventilation of the Servo ventilators; Maquet, Sweden). In this apparatus, esophageal electromyography monitoring can be performed even with the patient off the ventilator. Finally, there are no population reference values, which makes it difficult to use this measurement as an index of diagnosis of muscle weakness.

How to measure

The received signals are amplified and filtered, and this can be adjusted and will depend on the characteristics of the acquired signal. The most commonly employed method is electromyography with electrodes over the muscle, after the region has been thoroughly cleaned to improve the transmission of the electrical signal.^(^ 59 ^)^ It is also possible to use needles inserted intramuscularly, thereby obtaining a signal that is less noisy and more representative of a particular muscle activity. In obese individuals, needle electromyography of the abdominal muscles has a more significant result than that obtained with surface electrode electromyography. Often, needle insertion is ultrasound guided in order to prevent bleeding or perforation of other organs.^(^ 60 ^,^ 61 ^)^ Finally, it is also possible to use esophageal electromyography, in which one seeks to study the activity of the crural diaphragm by positioning the electrodes 1 to 3 cm above the esophageal-gastric junction.^(^ 62 ^,^ 63 ^)^

Clinical applicability

Electromyography is a reliable method for continuous monitoring, especially of certain respiratory muscles, such as abdominal expiratory muscles and inspiratory accessory muscles, provided that care is taken in the technical preparation. Its major use is in monitoring the same individual continuously, because absolute values do not allow comparisons between individuals. Surface electromyography is used for qualitative assessment of recruitment of inspiratory accessory muscles and abdominal expiratory muscles. When this assessment needs to be more specific or quantitative (usually for research purposes), the method used is needle electromyography. In addition to being used for research purposes, diaphragm electromyography is used as a guide in the NAVA mode of ventilation.

## Final considerations

Respiratory muscle impairment is present not only in respiratory diseases but also in various other diseases, and its proper assessment depends on the use of appropriate tests. Noninvasive volitional tests are still the most commonly used tests in clinical practice, because they are more widely known and because of their ease of use in different centers. However, patients highly suspected of having ventilatory muscle weakness and with difficulty understanding these tests should undergo additional assessment with more invasive, non-volitional tests, although they are not readily available and, to date, they have been mostly used in research centers.

## References

[1] Laghi F, Tobin MJ (2003). Disorders of the respiratory muscles. Am J Respir Crit Care Med.

[2] Dyspnea (1999). Mechanisms, assessment, and management: a consensus statement. American Thoracic Society. Am J Respir Crit Care Med.

[3] American Thoracic Society/European Respiratory Society (2002). ATS/ERS Statement on respiratory muscle testing. Am J Respir Crit Care Med.

[4] Neder JA, Andreoni S, Lerario MC, Nery LE (1999). Reference values for lung function tests. II. Maximal respiratory pressures and voluntary ventilation. Braz J Med Biol Res.

[5] Black LF, Hyatt RE (1969). Maximal respiratory pressures: normal values and relationship to age and sex. Am Rev Respir Dis.

[6] Black LF, Hyatt RE (1971). Maximal static respiratory pressures in generalized neuromuscular disease. Am Rev Respir Dis.

[7] De Troyer A, Borenstein S, Cordier R (1980). Analysis of lung volume restriction in patients with respiratory muscle weakness. Thorax.

[8] Caruso P, Friedrich C, Denari SD, Ruiz SA, Deheinzelin D (1999). The unidirectional valve is the best method to determine maximal inspiratory pressure during weaning. Chest.

[9] Yang KL, Tobin MJ (1991). A prospective study of indexes predicting the outcome of trials of weaning from mechanical ventilation. N Engl J Med.

[10] Sociedade Brasileira de Pneumologia e Tisiologia (2002). Diretrizes para Testes de Função Pulmonar. J Pneumol.

[11] Koulouris N, Mulvey DA, Laroche CM, Green M, Moxham J (1988). Comparison of two different mouthpieces for the measurement of Pimax and Pemax in normal and weak subjects. Eur Respir J.

[12] Truwit JD, Marini JJ (1992). Validation of a technique to assess maximal inspiratory pressure in poorly cooperative patients. Chest.

[13] Caruso P, Denari SD, Ruiz SA, Bernal KG, Manfrin GM, Friedrich C (2005). Inspiratory muscle training is ineffective in mechanically ventilated critically ill patients. Clinics (Sao Paulo).

[14] Martin AD, Smith BK, Davenport PD, Harman E, Gonzalez-Rothi RJ, Baz M (2011). Inspiratory muscle strength training improves weaning outcome in failure to wean patients: a randomized trial. Crit Care.

[15] Koulouris N, Vianna LG, Mulvey DA, Green M, Moxham J (1989). Maximal relaxation rates of esophageal, nose, and mouth pressures during a sniff reflect inspiratory muscle fatigue. Am Rev Respir Dis.

[16] Héritier F, Rahm F, Pasche P, Fitting JW (1994). Sniff nasal inspiratory pressure. A noninvasive assessment of inspiratory muscle strength. Am J Respir Crit Care Med.

[17] Katagiri M, Abe T, Yokoba M, Dobashi Y, Tomita T, Easton PA (2003). Neck and abdominal muscle activity during a sniff. Respir Med.

[18] Steier J, Kaul S, Seymour J, Jolley C, Rafferty G, Man W (2007). The value of multiple tests of respiratory muscle strength. Thorax.

[19] Laroche CM, Mier AK, Moxham J, Green M (1988). The value of sniff esophageal pressures in the assessment of global inspiratory muscle strength. Am Rev Respir Dis.

[20] Uldry C, Fitting JW (1995). Maximal values of sniff nasal inspiratory pressure in healthy subjects. Thorax.

[21] Polkey MI, Moxham J (2001). Clinical aspects of respiratory muscle dysfunction in the critically ill. Chest.

[22] Araújo PR, Resqueti VR, Nascimento J, Carvalho Lde A, Cavalcanti AG, Silva VC (2012). Reference values for sniff nasal inspiratory pressure in healthy subjects in Brazil: a multicenter study. J Bras Pneumol.

[23] Chaudri MB, Liu C, Watson L, Jefferson D, Kinnear WJ (2000). Sniff nasal inspiratory pressure as a marker of respiratory function in motor neuron disease. Eur Respir J.

[24] Stefanutti D, Benoist MR, Scheinmann P, Chaussain M, Fitting JW (2000). Usefulness of sniff nasal pressure in patients with neuromuscular or skeletal disorders. Am J Respir Crit Care Med.

[25] Uldry C, Janssens JP, de Muralt B, Fitting JW (1997). Sniff nasal inspiratory pressure in patients with chronic obstructive pulmonary disease. Eur Respir J.

[26] Lofaso F, Nicot F, Lejaille M, Falaize L, Louis A, Clement A (2006). Sniff nasal inspiratory pressure: what is the optimal number of sniffs?. Eur Respir J.

[27] Baydur A, Behrakis PK, Zin WA, Jaeger M, Milic-Emili J (1982). A simple method for assessing the validity of the esophageal balloon technique. Am Rev Respir Dis.

[28] Hamnegåard CH, Wragg S, Kyroussis D, Mills G, Bake B, Green M (1995). Mouth pressure in response to magnetic stimulation of the phrenic nerves. Thorax.

[29] Hughes PD, Polkey MI, Kyroussis D, Hamnegard CH, Moxham J, Green M (1998). Measurement of sniff nasal and diaphragm twitch mouth pressure in patients. Thorax.

[30] de Bruin PF, Watson RA, Khalil N, Pride NB (1998). Use of mouth pressure twitches induced by cervical magnetic stimulation to assess voluntary activation of the diaphragm. Eur Respir J.

[31] Baydur A, Cha EJ, Sassoon CS (1987). Validation of esophageal balloon technique at different lung volumes and postures. J Appl Physiol (1985).

[32] Polkey MI, Harris ML, Hughes PD, Hamnegärd CH, Lyons D, Green M (1997). The contractile properties of the elderly human diaphragm. Am J Respir Crit Care Med.

[33] de Troyer A, Yernault JC (1980). Inspiratory muscle force in normal subjects and patients with interstitial lung disease. Thorax.

[34] Laporta D, Grassino A (1985). Assessment of transdiaphragmatic pressure in humans. J Appl Physiol (1985).

[35] Akoumianaki E, Maggiore SM, Valenza F, Bellani G, Jubran A, Loring SH (2014). The application of esophageal pressure measurement in patients with respiratory failure. Am J Respir Crit Care Med.

[36] Beda A, Güldner A, Carvalho AR, Zin WA, Carvalho NC, Huhle R (2014). Liquid- and air-filled catheters without balloon as an alternative to the air-filled balloon catheter for measurement of esophageal pressure. PLoS One.

[37] Chiumello D, Gallazzi E, Marino A, Berto V, Mietto C, Cesana B (2011). A validation study of a new nasogastric polyfunctional catheter. Intensive Care Med.

[38] Stell IM, Tompkins S, Lovell AT, Goldstone JC, Moxham J (1999). An in vivo comparison of a catheter mounted pressure transducer system with conventional balloon catheters. Eur Respir J.

[39] Wragg S, Aquilina R, Moran J, Ridding M, Hamnegard C, Fearn T (1994). Comparison of cervical magnetic stimulation and bilateral percutaneous electrical stimulation of the phrenic nerves in normal subjects. Eur Respir J.

[40] Similowski T, Fleury B, Launois S, Cathala HP, Bouche P, Derenne JP (1989). Cervical magnetic stimulation: a new painless method for bilateral phrenic nerve stimulation in conscious humans. J Appl Physiol (1985).

[41] Man WD, Moxham J, Polkey MI (2004). Magnetic stimulation for the measurement of respiratory and skeletal muscle function. Eur Respir J.

[42] Lerolle N, Diehl JL (2011). Ultrasonographic evaluation of diaphragmatic function. Crit Care Med.

[43] Houston JG, Fleet M, Cowan MD, McMillan NC (1995). Comparison of ultrasound with fluoroscopy in the assessment of suspected hemidiaphragmatic movement abnormality. Clin Radiol.

[44] Matamis D, Soilemezi E, Tsagourias M, Akoumianaki E, Dimassi S, Boroli F (2013). Sonographic evaluation of the diaphragm in critically ill patients. Technique and clinical applications. Intensive Care Med.

[45] Lerolle N, Guérot E, Dimassi S, Zegdi R, Faisy C, Fagon JY (2009). Ultrasonographic diagnostic criterion for severe diaphragmatic dysfunction after cardiac surgery. Chest.

[46] Gottesman E, McCool FD (1997). Ultrasound evaluation of the paralyzed diaphragm. Am J Respir Crit Care Med.

[47] Summerhill EM, El-Sameed YA, Glidden TJ, McCool FD (2008). Monitoring recovery from diaphragm paralysis with ultrasound. Chest.

[48] Boon AJ, Harper CJ, Ghahfarokhi LS, Strommen JA, Watson JC, Sorenson EJ (2013). Two-dimensional ultrasound imaging of the diaphragm: quantitative values in normal subjects. Muscle Nerve.

[49] Boussuges A, Gole Y, Blanc P (2009). Diaphragmatic motion studied by m-mode ultrasonography: methods, reproducibility, and normal values. Chest.

[50] Testa A, Soldati G, Giannuzzi R, Berardi S, Portale G, Gentiloni Silveri N (2011). Ultrasound M-mode assessment of diaphragmatic kinetics by anterior transverse scanning in healthy subjects. Ultrasound Med Biol.

[51] Rahn H, Otis A, Fenn WO (1946). The pressure-volume diagram of the thorax and lung. Fed Proc.

[52] Coelho CM, Carvalho RM, Gouvêa DS, Novo JM (2012). Comparisons among parameters of maximal respiratory pressures in healthy subjects. J Bras Pneumol.

[53] Irwin RS, Boulet LP, Cloutier MM, Fuller R, Gold PM, Hoffstein V (1998). Managing cough as a defense mechanism and as a symptom. A consensus panel report of the American College of Chest Physicians. Chest.

[54] Khamiees M, Raju P, DeGirolamo A, Amoateng-Adjepong Y, Manthous CA (2001). Predictors of extubation outcome in patients who have successfully completed a spontaneous breathing trial. Chest.

[55] Man WD, Kyroussis D, Fleming TA, Chetta A, Harraf F, Mustfa N (2003). Cough gastric pressure and maximum expiratory mouth pressure in humans. Am J Respir Crit Care Med.

[56] Kyroussis D, Mills GH, Polkey MI, Hamnegard CH, Koulouris N, Green M (1996). Abdominal muscle fatigue after maximal ventilation in humans. J Appl Physiol (1985).

[57] Kyroussis D, Polkey MI, Mills GH, Hughes PD, Moxham J, Green M (1997). Simulation of cough in man by magnetic stimulation of the thoracic nerve roots. Am J Respir Crit Care Med.

[58] Suzuki J, Tanaka R, Yan S, Chen R, Macklem PT, Kayser B (1999). Assessment of abdominal muscle contractility, strength, and fatigue. Am J Respir Crit Care Med.

[59] Merletti R, Botter A, Troiano A, Merlo E, Minetto MA (2009). Technology and instrumentation for detection and conditioning of the surface electromyographic signal: state of the art. Clin Biomech (Bristol, Avon).

[60] Daube JR, Rubin DI (2009). Needle electromyography. Muscle Nerve.

[61] Rubin DI (2012). Needle electromyography: basic concepts and patterns of abnormalities. Neurol Clin.

[62] Luo YM, Moxham J, Polkey MI (2008). Diaphragm electromyography using an oesophageal catheter: current concepts. Clin Sci (Lond).

[63] Luo YM, Polkey MI, Johnson LC, Lyall RA, Harris ML, Green M (1998). Diaphragm EMG measured by cervical magnetic and electrical phrenic nerve stimulation. J Appl Physiol (1985).

[64] Criswell E (2010). Cram's Introduction to Surface Electromyography.

